# Effect and Molecular Mechanisms of Traditional Chinese Medicine on Regulating Tumor Immunosuppressive Microenvironment

**DOI:** 10.1155/2015/261620

**Published:** 2015-06-16

**Authors:** Qiujun Guo, Jie Li, Hongsheng Lin

**Affiliations:** ^1^Department of Oncology, Guang'anmen Hospital, China Academy of Chinese Medical Sciences, No. 5 Beixiange, Xicheng District, Beijing 100053, China; ^2^Beijing University of Chinese Medicine, No. 11 North Third Ring Road East, Chaoyang District, Beijing 100029, China

## Abstract

Traditional Chinese medicine (TCM) is an important complementary strategy for treating cancer in China. The mechanism is related to regulating the internal environment and remodeling the tumor immunosuppressive microenvironment (TIM). Herein we illustrate how TIM is reformed and its protumor activity on promoting tumor cell proliferation, angiogenesis and lymphangiogenesis, tumor invasion, and the oncogenicity of cancer stem cells. Furthermore we summarize the effects and mechanism of TCM on regulating TIM via enhancing antitumor immune responses (e.g., regulating the expression of MHC molecules and Fas/FasL, attenuating cancerigenic ability of cancer stem cells) and remolding immunosuppressive cells (e.g., reversing immune phenotypes of T lymphocytes and tumor associated macrophages, promoting dendritic cells mature, restraining myeloid derived suppressor cells function, and regulating Th1/Th2 factors). We also reveal the bidirectional and multitargeting functions of TCM on regulating TIM. Hopefully, it provides new theoretical basis for TCM clinical practice in cancer treatment and prevention.

## 1. Introduction

Chronic inflammation and immune suppression are the two core characteristics of the tumor microenvironment. It has been proven that chronic inflammation plays an important role in tumorigenesis and development; for instance inflammatory large intestine disease leads to colorectal cancer, H. Pylori infection breeds gastric cancer, and hepatitis B and C virus infection causes hepatocellular carcinoma (HCC). However, immune cells in tumor microenvironment promote tumor progression on the other hand: they constitute tumor immunosuppressive microenvironment (TIM) and alleviate tumor immune escape and tumorigenesis. Harmful stimulating factors such as hypoxia, acid environment, hyperosmosis, and inflammatory cytokines in tumor microenvironment facilitate the formation of TIM. According to the theory of tumor immunoediting, in TIM released tumor cells and immunosuppressive factors remodel the phenotype of immune cells, which decreases its antitumor function; meanwhile, remodeled immune cells “resculpture” tumor cells and make them become of low immunogenicity and might favor immune escape of tumor cells [1, 2]. Besides these, immune suppressive cells in TIM also bring out angiogenesis and lymphangiogenesis, playing a vital role in tumor development and metastasis. Thus, TIM is considered as a novel target for cancer treatment.

Traditional Chinese medicine (TCM) is a very important tumor treatment strategy in China [3]. It is accepted that TCM can reduce the toxicity of chemotherapy and radiotherapy, enhance the antitumor effect of these therapies, alleviate tumor-induced clinical symptoms and cancer pains, and prolong the survival time of postoperational and advanced stage cancer patients [4]. Though the effect mechanism of TCM is not very clear, increasing data has shown that it may relate with its action on regulating tumor immune environment, the novel target according to the theory of immunoediting [5, 6]. In this paper, we will introduce the formation and function of TIM and summarize recent researches of TCM on regulating it.

## 2. Tumor and Its Matrix Cells Constitute Tumor Immunosuppressive Microenvironment

Tumor microenvironment is a complex compound, including tumor cells, stromal cells, extracellular matrix, cellular factors, and chemokines. The microenvironment plays a pivotal role in the process of cancer development and metastasis. Downregulating the immune function and combined with extracellular matrix proteins and matrix-degrading enzymes, stromal cells (e.g., angiogenic cells, immune cells, and tumor associated fibroblasts) and cell factors they produced encompass tumor cells and form the tumor immunosuppressive microenvironment [7].

In TIM, tumor cells do not express classical MHC I molecules but express nonclassical MHC molecules, leading to the immunosuppression and tumor progression [8]. Meanwhile, tumor cells express a variety of immunosuppressive factors, such as IL-10, IL-6, and TGF-*β*, reversing immune cells to immunosuppressive phenotypes, for example, regulatory T cells, regulatory B cells, tumor associated macrophages (TAMs), regulatory dendritic cells, and myeloid derived suppressor cells (MDSCs). These immunosuppressive cells interact with each other and increasingly proliferate and express immunosuppressive and protumor factors. Moreover, they consume proinflammatory factors (IL-2, TNF-*α*), promote T cells apoptosis, and reduce the antitumor activity of NK cells. They produce matrix metalloproteinases (MMPs) and vascular endothelial growth factors (VEGF) as well, promoting angiogenesis and tumor invasion [9–12]. All these tumorigenesis responses give negative feedback to immunocytes, breeding a vicious circle for tumor treating.

## 3. Immune Suppressive Cells in TIM Promote Tumorigenesis and Progression

### 3.1. Promoting Tumor Cell Proliferation

Studies have proven the close relationship between TIM and tumorigenesis in different kinds of cancer diseases [13, 14]. When turning to basic researches, Fritz et al. [15] found that macrophages produce IGF-1 which directly stimulates neoplastic proliferation through Erk and Akt activation. Munari et al. [16] demonstrated that gastric lymphoma-infiltrating macrophages highly produced APRIL, which was regarded as a novel cytokine crucial in sustaining B cell proliferation and causing a mucosa associated lymphoid tissue B cell lymphoma proliferation. When coculturing with breast cancer cells, IL-4-activated macrophage transported microRNAs from itself to breast cancer cells, and one of microRNAs, miR-223, promoted the invasion of breast cancer cells via the Mef2c-*β*-catenin pathway [17]. These studies conclude and inside provide evidences that immune suppressive cells in TIM promote tumor cells multiplication.

### 3.2. Promoting Angiogenesis and Lymphangiogenesis

Tumor hematal and lymphatic vessels are important in tumor progression and serve as paths and tubes for nutrients and metabolites transportation, promoting tumor cells growth and metastasis. TIM plays a vital role in the process of angiogenesis and lymphangiogenesis. In tumor hypoxic microenvironment, tumor cells, tumor associated macrophages, dendritic cells, myeloid derived suppressor cells, and neutrophil cells secrete a variety of proangiogenesis and prolymphangiogenesis factors (e.g., VEGF, VEGF-C, MMP-9, TGF-*β*, and COX-2) [18, 19]. In recent years, studies also found a subtype of TAMs, called TIE2-expressing macrophages (TEM). TEMs overexpress angiopoietin-2 (ANG2) and mediate a crosstalk with vascular endothelial cells via Ang2-TIE2 pathway to promote angiogenesis [20]. Moreover, TAMs could transdifferentiate to lymphatic endothelial cells and, as endothelial progenitor cells, take part in lymphangiogenesis in tumor tissues under certain conditions [21]. Thereby, TIM is directly involved in the formation of vessels in tumors.

### 3.3. Promoting Tumor Invasion

The invasive ability of tumor cells is the basis of tumor local invasion and distant metastasis, and prior to this is the remolding of the extracellular matrix. TAM, Th-17 cells, and DCs in TIM produce MMPs and TGF-*β* to dissolve extracellular collagens, so as to remold the extracellular matrix. TAMs used both the mesenchymal mode requiring MMPs and the amoeboid migration mode to infiltrate tumor cell spheroids and promote MMP-independent invasion by tumor cells [22]. Epithelial mesenchymal transition (EMT) is the morphological change process of tumor cells invasion and metastasis [23]. TAMs could mediate EMT of tumor cells and promote the tumor progression through the TLR4/IL-10 signaling pathway [24]. By activating TGF-*β*, EGF, and HGF signaling pathway, MDSCs induced EMT as well [25]. Hence, TIM makes it easier for tumor invasion via both affecting it straightly and mediating EMT.

### 3.4. Promoting the Oncogenicity of Cancer Stem Cell

**Figure 1:**
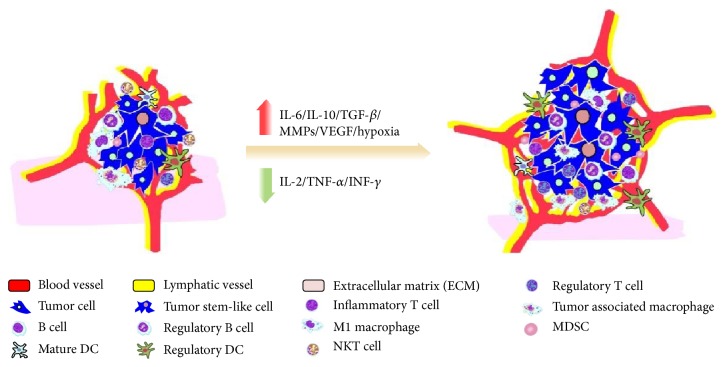
Tumor immunosuppressive microenvironment promotes tumorigenesis and progression.

Cancer stem-like cell (CSC) is a hotspot of the tumor research in recent years. It is widely thought that CSC is a tumorigenesis and metastasis “seed cell” and the “springhead” of tumor immune escape. CSC has a continuous self-renewal and proliferation ability, and it differentiates incompletely, resisting radiation and chemotherapy and producing immune suppressive factors. Losing normal exogenous apoptosis signaling pathways, CSC strongly shows the resistance to apoptosis functions [26, 27]. Immune suppressive cells in TIM also participate in regulating CSC function. Yang et al. [28] found that TAMs mediate tumorigenesis through regulation of breast CSCs and promote CSC-like phenotypes in murine breast cancer cells by upregulating their expression of Sox-2. TAM markers are associated with cancer stem cell marker in oral squamous cell carcinoma [29]. MDSCs had similar features that triggered microRNA101 expression in cancer cells and increased cancer cell stemness and increasing metastatic and tumorigenic potential targeted stem cell core genes [30]. In conclusion, TIM enhances CSC progression in mutual effect (Figure 1).

The hypoxia environment brings TIM increasingly producing immune suppressive and angiogenesis factors (e.g., IL-6, IL-10, TGF-*β*, MMPs, and VEGF) and downregulating inflammatory cytokines such as IL-2, TNF-*α*, and INF-gamma, promoting immune suppressive cells proliferation and CSCs growth and inhibiting antitumor cells. Thus, TIM leads to tumorigenesis, angiogenesis, lymphangiogenesis, and tumor ECM remolding and causes tumor progression and metastasis.

## 4. TCM Enhances Tumor Immune Responses

### 4.1. TCM Promotes Classic MHC Molecules Expression

MHC, also called human leukocyte antigen (HLA) in human tissues, is the important immunological recognition molecule in the process of tumor immune response. Classic MHC molecule can be divided into two subgroups: MHC class I and MHC class II. MHC I have a vital role in presenting tumor antigens to T cell receptor. After identifying the tumor antigen peptide and MHC class I molecule, the cytotoxic T cell (CTL) is activated and launched a series of cytolysis reactions to kill tumor cells. MHC II presents tumor antigens to CD4^+^T helper cells, leading to cellular mediated immune response. However, both immune and malignant cells in the tumor microenvironment do not express typical MHC molecules. They downregulate MHC class I expression, overexpress nonclassical HLA such as HLA-G, HLA-E, and HLA-F, which have been recently proven to be correlated with poor clinical outcome, and escape from T and NK cell-mediated recognition [8, 31].

Studies have shown that TCM can upregulate MHC molecules in tumor microenvironment. Li et al. [32] explored the effect of Invigorating Spleen and Detoxification Decoction (ISD) (Radix Codonopsis,* Poria*, Rhizoma Atractylodis Macrocephalae, Radix Glycyrrhizae, Radix Bupleuri, Rhizoma Curcumae, and* Herba Scutellariae barbatae*) on MHC molecules in the rat liver cancer tissue and found that ISD could enhance the expression of MHC I and MHC II and prolong the rat survival time. Besides the effects on tumor cells, TCM could also increase MHC expression in immune cells.* Anoectochilus formosanus* is a medicinal herb in Asia and extracts of* A. formosanus* have been reported to possess antitumor activities. Kuan et al. [33] found that* A. formosanus* could stimulate the MHC II expression. In vitro experiment showed Fei Liu Ping Extractum (FLP) (Radix Panacis Quinquefolii,* Cordyceps*, Radix Astragali, Radix Codonopsis, Radix Glehniae, Radix Ophiopogonis, Herba Agrimoniae,* Polygonum bistorta* L.,* Thlaspi arvense* Linn.,* Hedyotis diffusa*, Semen Juglandis, Semen* Armeniacae amarum*, and Bulbus Fritillariae Cirrhosae) could upregulate the expression of MHC II of dendritic cells and improve the body function of antitumor immunological surveillance [34]. These results suggest that TCM could enhance tumor antigen-presenting ability by upregulating classic MHC expression in both tumor and immune cells.

### 4.2. TCM Induces Tumor Cells Apoptosis via Fas/FasL Pathway

Fas and its natural ligand FasL are molecules expressed on cellular membranes. The activation of Fas/FasL pathway plays an important role in cell apoptosis. Physiologically, cytotoxic T lymphocytes (Fas^low^FasL^high^) express FasL and combine with the Fas expressed by target cells (Fas^high^FasL^low^), resulting in the trimerization and activation of the Fas receptor, and then mediate target cells apoptosis. Unlike normal target cells, tumor cells express nonfunctional Fas or low quantity of Fas; meanwhile overexpression of FasL promotes tumor cells immune escape by preventing the combination with cytotoxic T cells and induces CTL apoptosis [35].

Our previous study showed that TCM herbal medicine Yang Wei Kang Liu Granule (YWKL) (Radix Astragali, Radix Ginseng,* Hedyotis diffusa*, Yunnan Manyleaf Paris Rhizome, Radix Notoginseng, Radix Paeoniae Rubra, and Hematoxylon) could increase FasL expression and downregulate Fas expression in T lymphocytes of gastric cancer patients, which indicates YWKLF may inhibit cancer by inducing apoptosis [36]. Our subsequent studies established MGC-803 stomach cancer cell model in vitro and showed FasL mRNA in MGC-803 declined significantly after treatment by YWKL [37]. These mean YWKL may enhance cancer cells' sensitivity to immune response cells like CTL and lead to tumor cells apoptosis by regulating Fas/FasL pathway.

### 4.3. TCM Attenuates Oncogenicity of CSCs

Cancer stem-like cells (CSCs) are a more malignant composition in tumor tissues inhibiting immune response. Researches have demonstrated CSCs take part in carcinogenesis and therapy resistance in kinds of tumors [38–41]. CSCs rarely express immune recognition molecules such as HLA-DR and costimulatory molecules such as CD80 and CD86. Furthermore, laboratory researches show CSC tumor spheres lowly express Fas and highly express membrane complement regulatory proteins and FoxP3 [42]. All these findings indicate CSCs prevent antitumor immune responses and promote immunosuppressive microenvironment formation. Thus, it is rational to target CSCs to reverse tumor immune suppression.

**Figure 2:**
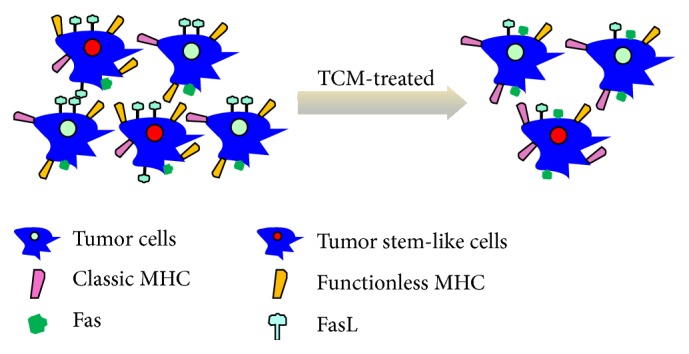
TCM enhances tumor cells immune responses.

Some medicinal herbs have been proved to have anti-CSC ability. Chang et al. [43] cultured the hMG63-derived cancer stem cells in permissive microenvironments for stem cell differentiation and found Bufalin inhibited the proliferation and sphere formation of cancer stem cells. Zhang et al. [44] found that Huaier (*Trametes robiniophila* Murr.) aqueous extract had significant efficacy on inhibiting spheroid formation (*P* < 0.05) and reduced the aldehyde dehydrogenase (ALDH) positive cell population in colorectal primary cancer cells (*P* < 0.05). Further study revealed Huaier extract downregulated the Wnt/*β*-catenin pathway, which is one of the critical pathways demonstrated to mediate the self-renewal of CSCs. Honokiol is an extract from medicinal herb Cortex Magnoliae Officinalis. Research showed that it can thwart tumor growth [45]. Recently, Ponnurangam et al. [46] found the ability of Honokiol to enhance the sensitivity of colon CSCs to ionizing radiation is by inhibiting the *γ*-secretase complex and the Notch signaling pathway. These findings indicate that Chinese medicine may reduce CSCs and weaken tumor immune resistance as well (Figure 2).

While being treated by Chinese medicine, the percentage of CSCs is decreased; Classic MHC and Fas molecules are expressed more on tumor cytomembranes, which lead to tumor cells having lower malignant degree and easier to be recognized and killed by the immune system.

## 5. TCM Reverses the Immunosuppressive Phenotype and Regulates the Antitumor Functions of Immune Cells

**Figure 3:**
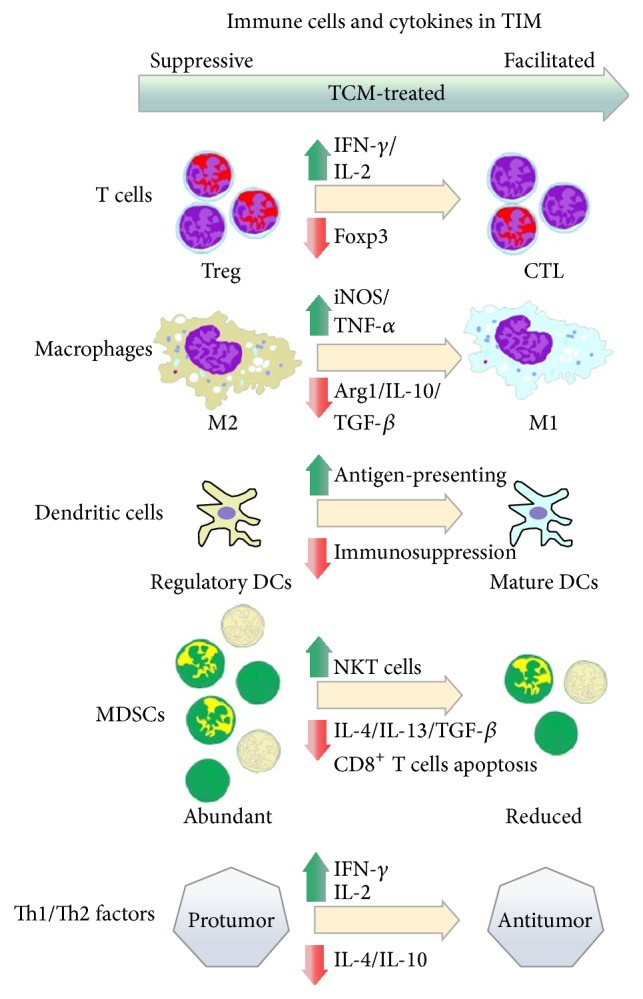
Molecular mechanism of TCM on regulating immune cells in TIM.

Immune cells show various functions and phenotypes and play multiple roles in antitumor immunity and protumorigenesis, which are determined and influenced by tumor immune microenvironment. TCM herbs indicate a biphasic regulation on tumor cells phenotype to enhance antitumor immune responses: increasing proinflammatory phenotypic antitumor activity while reversing and remolding the suppressive function (phenotype) of immunocytes (Figure 3).

### 5.1. TCM Enhances T lymphocyte Antitumor Abilities

T lymphocytes are notably involved in tumor adaptive immune cells but also have been found participating in tumor immune suppression in different subtypes [47]. T cells express specific recognition and signal transduction related to TCR/CD3 complex molecules. Based on their function and phenotypes, T cells are divided into three main subtypes: cytotoxic T cells (CD3^+^CD8^+^), helper T cells (CD3^+^CD4^+^), and regulatory T cells (CD3^+^CD4^+^CD25^+^). CD4^+^T cells produce IL-2 and IL-15 which enhance the activation of CD8^+^T cells, maintain the activity of CTL, and also activate innate immune cells such as natural kill cells and dendritic cells. Meanwhile, part of CD4^+^T cells has the ability to kill tumor cells directly. CD8^+^T cells release perforin and particle enzymes to kill and dissolve tumor cells and lead to target cells apoptosis through Fas/FasL pathway. CD4^+^CD25^+^T cell (Treg) is an immune regulating subtype which is regarded to play immunosuppressive role in tumor microenvironment. Tregs produce quantity of immune suppressive factors, for example, IL-10 and TGF-*β*, compete with response T cells for consuming IL-2, affect metabolism of other T cells, and downregulate the stimulation of dendritic cells [48]. Thus, it is the key to regulating tumor immune microenvironment via enhancing antitumor efficacy of T cells and inhibiting Treg cell and its function.

It is notable that TCM may adjust immune function by targeting T cells. Feiyanning Decoction (FYN) (Radix Astragali, Rhizoma Atractylodis Macrocephalae, Pseudobulbus Cremastrae seu Pleiones,* Salvia Chinensis* Benth.,* Paris polyphylla*, Corium Bufonis, Nidus Vespae, Rhizoma Polygonati, Fructus Corni, Herba Epimedii, Fructus Ligustri Lucidi, and* Ganoderma*) is an antitumor compound prescription of TCM, which has been proven effective in the clinical research [49]. Recent study in Lewis lung carcinoma bearing mice model showed FYN's effect on Tregs. It was found that the numbers of CD4^+^CD25^+^ regulatory T cells in spleen, thymus, and tumor were lower in the FYN group than in the model group (*P* < 0.05). The expression of Foxp3 mRNA in spleen, thymus, and tumor was also significantly downregulated in the FYN group [50]. There are other studies that showed main ingredients of FYN such as Astragaloside significantly increased IL-2 and IFN-*γ* secretion of T cells and promoted T cells immune activity [51].

### 5.2. TCM Regulates M1/M2 Phenotypes of TAMs

Tumor associated macrophages (TAMs) derived from peripheral circulating monocytes occupy about 30%–50% of the total tumor stroma cells. After being recruited to the tumor microenvironment, TAMs differentiate into two polarized phenotypes, that is, the classic activation polarization (M1 phenotype) and the alternative activation polarization (M2 phenotype). Induced by IFN-*γ*, IL-6, and so forth, M1 macrophages display proinflammatory, antigen-presenting, and antitumor effects, through release of soluble enzyme, TNF, and IFN and activation of T cell immune responses to inhibit tumor cells. However, M2 macrophages, activated by IL-10, IL-13, and so forth, have an immune regulating and suppressive role via multiple ways to promote tumor progression. Recent studies indicated TAMs tend to M2 protumor phenotype through stimulating tumor cell proliferation, inhibiting tumor immune microenvironment, promoting matrix remodeling, and accelerating angiogenesis and lymphangiogenesis [52, 53]. Thus, TAMs are associated with tumor progression and metastasis.

Researchers reported TCM herbs could inhibit macrophages' inflammatory effect [54], and there are certain studies that showed TCM could switch the phenotype of TAMs from M2 to M1 during tumor progression.* Schisandra* polysaccharide (SCPP11) is an extract ingredient from herb medicine* Schisandra chinensis*, which has been used in TCM for centuries and proved to have antitumor activities. Recent study results showed that SCPP11 could increase the pinocytic activity of peritoneal macrophages in CTX-induced immunosuppression mice. Moreover, SCPP11 significantly increased immunoglobulin levels and cytokines levels in vivo and induced RAW264.7 cells (a monocyte/macrophage cell line of mice for in vitro experiments) to secrete cytokines in vitro and RAW264.7 cells pretreated with SCPP11 significantly inhibited the proliferation of HepG-2 cells, via promoting the expression of both iNOS protein and iNOS and TNF-*α* mRNA [55]. These results showed that SCPP11 could enhance the antitumor effect of macrophages. Other researchers found five extracts (UM01, QH11, BNQM, GNCC, and DCXC) of* Cordyceps sinensis* could significantly increase cell proliferation and NO production of RAW264.7 cells [56]. Zhang et al. found that* Ganoderma atrum* polysaccharide (PSG-1) increased macrophage phagocytosis and the levels of cytokines and nitride oxide through TLR4-mediated NF-*κ*B and MAPK signaling pathways in S180 tumor bearing mice model [57]. Shenqi Fuzheng Injection (Radix Codonopsis, Radix Astragali) was indicated enhancing peritoneal macrophage phagocytosis in immunosuppressed mice as well [58]. Our studies found similar results that TCM decoction Fuzheng Jiedu Formula (FZJD) (Radix Astragali, Radix Codonopsis, Rhizoma Atractylodis Macrocephalae, Radix Polygoni Multiflori, Fructus Lycii, Yunnan Manyleaf Paris Rhizome,* Hedyotis diffusa*, and* Actinidia arguta* Planch.), including Radix Codonopsis and Radix Astragali, not only promoted macrophage phagocytosis but also enhanced M1/M2 and anti-/protumor factors of TAMs in tumor microenvironment. Specifically, FZJD reduced IL-10 and TGF-*β* expression and raised the ratio of iNOS/Arg1 which represents the M1/M2 proportion. All the findings above illustrate Chinese medicinal herbs could inhibit tumor cells by promoting macrophages antitumor function and reversing TAM phenotype M2 to M1.

### 5.3. TCM Enhancing Antigen-Presenting Function of Dendritic Cells (DCs)

Dendritic cells (DCs) are professional antigen-presenting cells, which activate initial T cell by presenting and delivering antigens and mediate acquired tumor immune cytotoxicity. However, recent studies found a subset of DCs in the tumor microenvironment inducing immunosuppression called regulatory DCs or tumor associated DCs (TADCs). These TADCs have a low ability to present antigen, induce T cells differentiating to Treg subtype, and consequently impair T cell-mediated tumor killing effects. Furthermore, TADCs lead to a decreasing ratio of Th cells and effective T cells apoptosis through a way such as reactive oxygen species, the indoleamine 2, 3-2 oxidase (IDO), and releasing immune suppressive factors [59]. TADCs are also involved in tumor angiogenesis, tumor cell proliferation, and invasion [60]. Thus, we can see from above that DCs have a similar polarization like macrophages (M1/M2). Regulating dendritic cells and enhancing their antigen-presenting function could be a possible way for TCM antitumor effects.

Zhang and Liu found that TCM with function of supplementing Pi and nourishing Shen could improve the inflammatory function of DCs in patients with chronic hepatitis B [61]. Our laboratory group explored the possible influence of TCM drug FLP on regulating DCs in peripheral blood, spleen, and tumor in mice with transplanted Lewis lung cancer. We found that the percentages of DCs (per thousand) in tumor bearing mice were increased to 2.55 ± 0.29 in peripheral blood and 2.70 ± 0.63 in spleen (*P* < 0.01) after FLP treatment [62]. Further studies showed FLP promoted DCs maturity, reversed DCs regulatory (immunosuppressive) phenotype, and increased DCs membrane MHC II, CD80, CD83, CD86, and CD40 expression. FLP also promoted the IL-12 secretion of DCs and enhanced the function of DC-LPAK tumor killing way [34, 63]. These results indicate TCM may have regulating effect on DCs.

### 5.4. TCM Restrains Myeloid Derived Suppressor Cells (MDSCs)

Myeloid derived suppressor cells (MDSCs) are a special kind of cell population which play essential role in malignant tumor immune suppression. The main immune inhibition effects of MDSCs reflect on suppressing T cells related immune responses and inducing T cell apoptosis, promoting inflammatory mediated tumor recurrence and metastasis. MDSCs are a group of heterogeneous cells, including immature macrophages, dendritic cells, and granulocytes. Pathological stimulations like malignant tumor and inflammation block immature bone marrow cells differentiation into mature cells, leading to the expansion of MDSCs and contributing to the negative regulation of tumor immune response. MDSCs facilitate angiogenesis by releasing VEGF, MMPs, TGF-*β*, and so forth and inhibiting T cell-mediated tumor acquired immune responses with overexpression of Arg1, iNOS, Ros, and so forth. The increasing IL-10 productivity of MDSCs restricts macrophages and DCs antitumor function as well [64].

Since 2007, we established the collaboration with the National Cancer Institute (NCI) Molecular Immunoregulation Laboratory and carried out series studies on the immune regulation effect of TCM. The results showed TCM herbs Radix Ginseng and Radix Notoginseng had a certain inhibitory effect on MDSCs phenotype and protumor functions [65]. Our recent studies found that the levels of MDSCs in breast cancer patient peripheral blood are positively associated with tumor progression and TCM decoction Shugan Jianpi Formula (Radix Bupleuri, Radix Paeoniae Alba, Spica Prunellae, Radix Curcumae, Holboellia Fargesii Reaub, Radix Astragali, Radix Notoginseng, and Radix Glycyrrhizae) (SGJP) had an inhibition function in MDSCs proliferation and could prevent MDSCs induced IL-4, IL-13, and TGF-*β* expression and CD8^+^T cells apoptosis. Meanwhile, SGJP enhanced and regulated inflammatory functions of NKT cells, which were associated with MDSCs regulation [66]. These above were addressed by Jak-Stats signal pathways and indicated TCM herbs affecting MDSCs.

### 5.5. TCM Regulates Th1/Th2 Immune Factors Secretion

Th1/Th2 immune factors also have a polarization of promoting antitumor immunocompetence and tumorigenesis ability. Th1 factors like IL-2, IL-6, TNF-*α*, and INF-*γ*, are linked to proinflammation, cytotoxic and cytophagic enhancement, and other antitumor functions, while Th2 factors, for example, IL-4, IL-10, and TGF-*β*, play a suppressive role in tumor immune microenvironment and promote tumor recurrence and metastasis.

Wei et al. [67, 68] found that TCM herb Radix Astragali (AG) and tetramethylpyrazine (TTMP) extracted from a medicinal herb* Ligusticum chuanxiong* could reverse predominance of Th2 cytokines in lung cancer patients. AG enhanced the levels of Th1 cytokine (IFN-*γ* and IL-2) as well as its transcript factor (T-bet) expression in culture supernatant and reduced those of Th2 cytokines in cultured peripheral blood mononuclear cells (PBMC) of lung cancer patients. TTMP could enhance supernatant concentration and gene expression levels of IFN-*γ*, IL-2, and T-bet but reduce those of type 2 cytokines (e.g., IL-4, IL-10). These results demonstrated that AG and TTMP could reverse the type 2 dominant status, which might offer an alternative therapeutic regime for lung cancer patients. Other studies of TCM compound decoctions, containing* Astragalus* composition, showed a similar result as well. Fuzheng Yiliu Decoction (FYD) (Ginseng,* Astragalus, Ganoderma lucidum, Angelica sinensis*, and* Lycium chinense*) remarkably inhibited proliferation and induced apoptosis of hepatoma cells at least by promoting the production of IL-2 and TNF-*α* in vivo [69]. Wang and Chen [70] observed the effect of Aidi injection (*Mylabris*, Radix Ginseng, Radix Astragali, and Radix et Caulis Acanthopanacis Senticosi) on peripheral blood expression of Th1/Th2 transcription factors and cytokines in patients with esophageal squamous cell carcinoma (ESCC) during radiotherapy. They found a Th2 toward drift phenomenon that, compared with the healthy control group, the expressions of Th1 type transcription factor T-bet and cytokines IFN-*γ*, IL-2 in ESCC patients were significantly lower, while expressions of Th2 type transcription factor GATA-3 and cytokines IL-4, IL-10 were significantly higher. When combined with intravenous dripping of Aidi injection during radiotherapy, the Th2 toward drift was inhibited. These results demonstrated that TCM herbs might reverse the Th2 predominant status, which is a probable alternative therapeutic regime in future.

Suppressive phenotypes of immune cells are regulated and reversed by TCM treating. Concretely, inflammatory T cells and NKT cells increasingly proliferate in contrast to the reducing quantity of regulatory T cells and MDSCs. Suppressive macrophages and DCs change their functions to antitumor effects, such as M2 to M1 phenotype reversing and DCs maturation. All these alterations are influenced and accompanied by increasing inflammatory factors expression and immune suppressive cytokines decreasing.

## 6. Difference between TCM and Modern Western Medicine in Cancer Immunotherapy

### 6.1. TCM Has Multitarget and Bidirectional Immunoregulation Effect

Based on the preliminary studies, the multitarget function of TCM compound on regulating TIM is assigned to its multicomponents. First, the compound is usually composed of more than one herb, and the herb often consists of various ingredients and each ingredient always contains different kinds of chemicals. There are some certain chemicals extracted from TCM herbs that are found to have immune regulating function: specifically,* Ganoderma lucidum* polysaccharides (GLPS), a kind of effective ingredient extracted from* Ganoderma lucidum* (*G. lucidum*), which has long been prescribed to prevent and treat various human diseases, particularly in China, Japan, and Korea. Some researches showed that GLPS had a potential role in cancer treatment; further studies suggested that the antitumor activities of GLPS are mediated by its immunoregulation effect. GLPS might act on immune-related cells as well as immune cells such as B lymphocytes, T lymphocytes, dendritic cells, macrophages, and natural killer cells [71].* Solanum nigrum* Linn., another effective TCM herb in cancer treatment, has been proven to have antitumor activity by enhancing the CD4^+^/CD8^+^ ratio of the T lymphocyte subpopulation. Razali et al. [72] named SN-ppF3 from* Solanum nigrum* Linn. polysaccharide that could significantly induce phagocytosis activity and stimulated the production of TNF-*α* and IL-6 and nitric oxide synthase expression of RAW264.7 cells.

Besides the ingredient found directly from the herbs, the postmetabolized ingredients of TCM compound may also play an immunoregulating role. Bae et al. [73] found that, after oral administration of BST204, a purified ginseng dry extract containing ginsenosides such as Rg1 and Rh1, only the S epimers, S-Rh2 and S-Rg3, could be determined in rat plasma. Studies also found that the oral bioavailability of ginsenoside Rg1 is very low in rats and ginsenosides might be metabolized by intestinal microflora before absorption into blood [74, 75]. These indicated that some ingredients in TCM herbs might be effective after being metabolized or some of the chemicals inactive in vitro could play roles in organisms after being metabolized. Continuing in depth studies, metabolite of ginsenosides compound K was found in suppressing the activation of the NF-*κ*B pathway and attenuating metastatic growth of hepatocellular carcinoma [76]. Another study showed that Rh2 metabolic modification ginsenoside* aglycone protopanaxadiol* (aPPD) activated in treating prostate cancer combined with docetaxel and was more effective than its prototype [77]. TCM herbs such as ginseng have been proved to have immune enhancement, and it is a possible hypothesis that their metabolites modulate immune microenvironments.

As mentioned above, due to its complicated ingredients TCM compound has a multitarget effect. It might act directly on various immune cells, while it can also play a role through regulating immune factors in different immune signal pathways [78]. Another important feature of TCM is its bidirectional effect. For instance, Radix Astragali has effects on both neoplasms (immune enhancement) and rheumatoid arthritis (immune suppression) [79, 80]. In addition, TIM brings out abnormal expression of signal pathways and cytokines; meanwhile the ratio of function-opposite immune cell phenotypes such as M1/M2 macrophages is also imbalanced in TIM, which is called disharmony according to TCM theory. TCM always affect multiple targets, and they could appropriately reverse an unbalanced state (immunologic derangement in TIM) to a relative harmony state.

### 6.2. Western Medicine Acts as Guided Missiles in Cancer Immunotherapy

The characteristic of Western medicine is focused on definite targets that means Western medicine has specific targets relatively visualized and detectable, just like a guided missile hit a target with a minimal error. These immunotherapies are guided into the following fields: (1) monoclonal antibodies against either tumor cells (bevacizumab [81], cixutumumab [82], rituximab [83], etc.) or immunosuppressive factors (tremelimumab [84], nivolumab [85], etc.); (2) tumor vaccines based on polypeptide (MAGE-A3 [86], etc.), DCs (belagenpumatucel-L [87], etc.), or other targets vaccines; (3) immune cells adoptive treatment, such as CIK [88]; (4) soluble tumor virus gene therapy [89], such as GC0070 and GL-ONC1. These indicate the convinced evidences and targets of Western medicine immunotherapy, but there are some problems and side effects, such as the dermatologic events associated with cixutumumab [90].

To make up for deficiencies of Western medicine specificity, it is feasible to introduce TCM in modern immunotherapies, such as the immune enhancement and side effects remission [91, 92]. Above all, it is benefit to patients that we combine the Western medicine (specificity) with TCM (multitarget and bidirectional regulating) in immunotherapies, and there is a promising field waiting us to study and explore.

## 7. Summary

**Figure 4:**
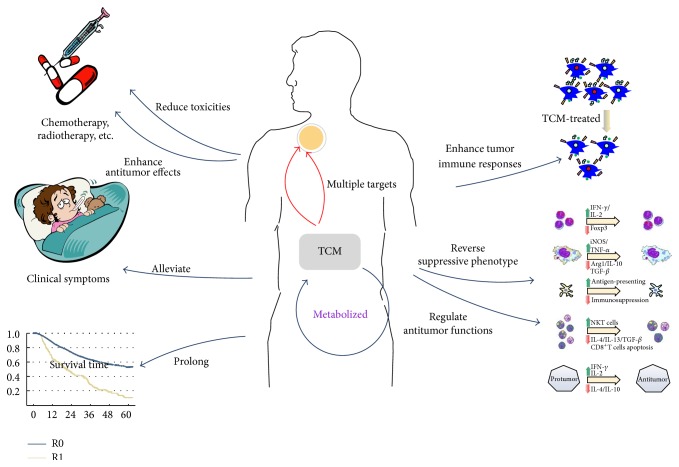
General functions and mechanisms of TCM on tumor therapies.

TCM plays an important role in cancer treatment in China via diversity ways (Figure 4). The formation and dynamic changes of TIM are a multifaceted process and are including multiple targets, different types of cells, and various signal pathways. Here we maintain that the TCM, both monomer and compound, has a certain effect on regulating TIM, and ways and points the TCM is affecting are diverse. More specifically, TCM herbs influenced not only tumor cells, but immune cells and cytokines as well as signal pathways [93, 94]. For the further analysis of these TCM herbs, we can see Radix Codonopsis, Radix Ginseng, Radix Astragali Preparata, Rhizoma Atractylodis Macrocephalae, Yunnan Manyleaf Paris Rhizome,* Hedyotis diffusa*, and so forth exist in kinds of TCM compound decoctions, which have different roles in regulating TIM; that is to say some of TCM herbs may have multiple effect on TIM. Treatment based on syndrome (ZHENG) differentiation is the characteristic and treatment guide of TCM. Studies showed tumor microenvironment differentiated under different TCM ZHENG models and had a relationship with treatment response to herbal medicine [95]. TCM herbs are cataloged according to their effect on certain TCM ZHENGs as Radix Codonopsis, Radix Ginseng, Radix Astragali Preparata, and Rhizoma Atractylodis Macrocephalae are tonified medicines to treat Qi deficiency ZHENG while Yunnan Manyleaf Paris Rhizome and* Hedyotis diffusa* relieve heat-toxin ZHENG in TCM pharmacology. TCM herbs are also considered to have dual-direction regulating effects on TIM as enhancing tumor cells antigenic responses and immune cells antitumor abilities while inhibiting the tumorigenesis functions. Above all, it is worth a further study to figure out the relationship and biochemical mechanism of different catalogs of TCM herbs in regulating TIM. With the rapid development of immunology, TCM effect on regulating TIM will play a more important role in tumor complementary and alternative therapies.

It has been accepted in China that TCM can reduce the toxicity and enhance the antitumor effect of chemotherapy and radiotherapy, alleviate tumor-induced clinical symptoms, and prolong the survival time of postoperational and advanced stage cancer patients [4]. Meanwhile, TCM regulates TIM by enhancing tumor immune responses, reverses suppressive phenotype of immune cells, and promotes their antitumor functions.

## Abbreviations

ANG: Angiopoietin
APRIL: A proliferation inducing ligand
COX-2: Cyclooxygenase-2
CSC: Cancer stem-like cell
DC: Dendritic cell
EGF: Epidermal growth factor
EMT: Epithelial mesenchymal transition
Erk: Extracellular regulated protein kinases
HCC: Hepatocellular carcinoma
HGF: Hepatocyte growth factor
HLA: Human leukocyte antigen
IGF-1: Insulin-like growth factor-1
IL: Interleukin
INF-*γ*: Gamma interferon
MDSC: Myeloid derived suppressor cell
MHC: Major histocompatibility complex
MMP: Matrix metalloproteinase
TAM: Tumor associated macrophage
TCM: Traditional Chinese medicine
TGF-*β*: Transforming growth factor-beta
TIM: Tumor immunosuppressive microenvironment
TNF-*α*: Tumor necrosis factor-alpha
VEGF: Vascular endothelial growth factor.
